# Supplementary material for: Dual Hyaluronic Acid and Folic Acid Targeting pH-Sensitive Multifunctional 2DG@DCA@MgO-Nano-Core–Shell-Radiosensitizer for Breast Cancer Therapy
Source: Cancers (Basel). 2021 Nov 7;13(21):5571. doi: 10.3390/cancers13215571 (PMC8583154; doi:10.3390/cancers13215571)
Supplement: Supplementary file 1 [file cancers-13-05571-s001.zip › cancers-1406449-supplementary.pdf]

cancers

MDPI

Supplementary Materials: Dual Hyaluronic Acid and Folic Acid Targeting pH-Sensitive Multifunctional 2DG@DCA@MgO-Nano-Core–Shell-Radiosensitizer for Breast Cancer Therapy

Mostafa A. Askar, Noura M. Thabet, Gharieb S. El-Sayyad, Ahmed I. El-Batal, Mohamed Abd El kodous, Omama E. El Shawi, Hamed Helal and Mohamed K. Abdel-Rafei

Figure 6f

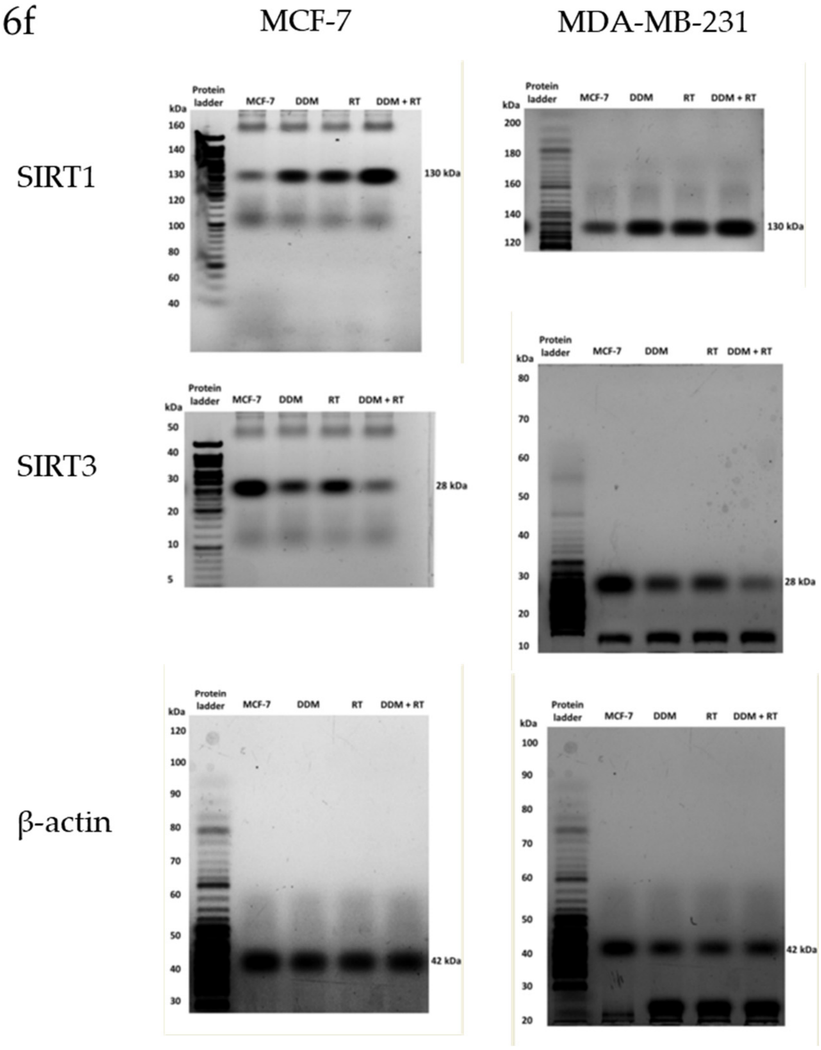

Figure 7a

MCF-7

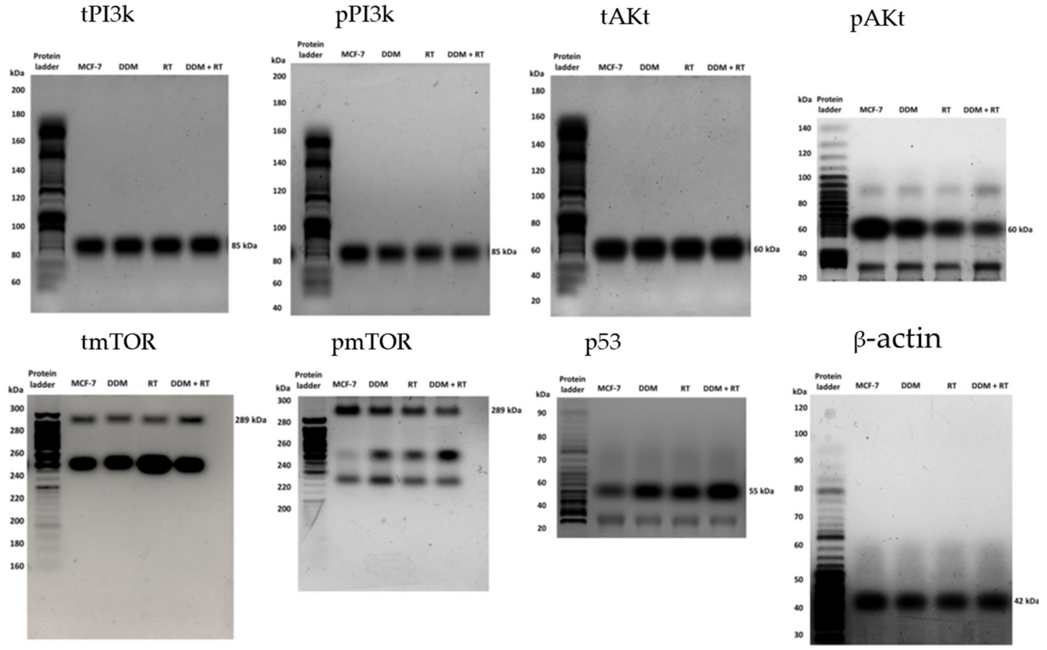

Figure 7d

MDA-MB-231

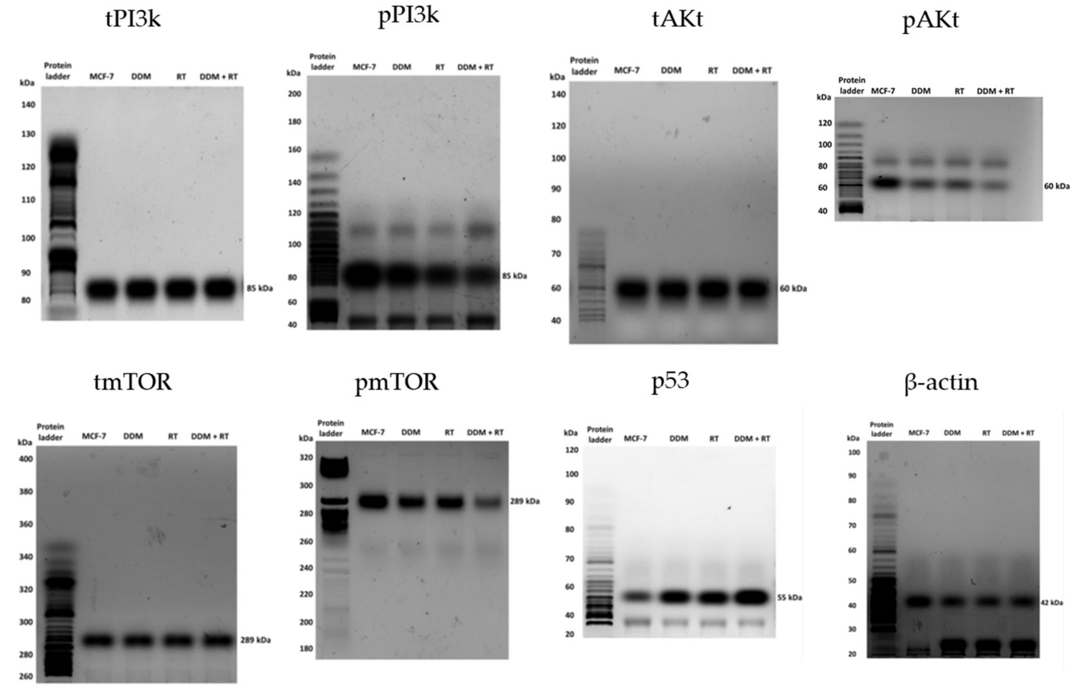

Figure S1. The uncropped Western blots.
